# Effects of dose scaling on delivery quality assurance in tomotherapy

**DOI:** 10.1120/jacmp.v13i1.3621

**Published:** 2012-01-05

**Authors:** Nathan Whitmore, Adrian Nalichowski, Jay Burmeister

**Affiliations:** ^1^ Wayne State University School of Medicine Department of Radiation Oncology, Gershenson Radiation Oncology Center Detroit MI 48201; ^2^ Karmanos Cancer Center, Gershenson Radiation Oncology Center Detroit MI 48201 USA

**Keywords:** tomotherapy, DQA, scaling, quality assurance

## Abstract

Delivery quality assurance (DQA) of tomotherapy plans is routinely performed with silver halide film which has a limited range due to the effects of saturation. DQA plans with dose values exceeding this limit require the dose of the entire plan to be scaled downward if film is used, to evaluate the dose distribution in two dimensions. The potential loss of fidelity between scaled and unscaled DQA plans as a function of dose scaling is investigated. Three treatment plans for 12 Gy fractions designed for SBRT of the lung were used to create DQA procedures that were scaled between 100% and 10%. The dose was measured with an ionization chamber array and compared to values from the tomotherapy treatment planning system. Film and cylindrical ion chamber measurements were also made for one patient for scaling factors of 50% to 10% to compare with the ionization chamber array measurements. The array results show the average gamma pass rate is ≥99% from 100% to 30% scaling. The average gamma pass rate falls to 93.6% and 51.1% at 20% and 10% scaling, respectively. Film analysis yields similar pass rates. Cylindrical ion chambers did not exhibit significant variation with dose scaling, but only represent points in the low gradient region of the dose distribution. Scaling the dose changes the mechanics of the radiation delivery, as well as the signal‐to‐noise ratio. Treatment plans which exhibit parameters that differ significantly from those common to DQA plans studied in this paper may exhibit different behavior. Dose scaling should be limited to the smallest degree possible. Planar information, such as that from film or a detector array, is required. The results show that it is not necessary to perform both a scaled and unscaled DQA plan for the treatment plans considered here.

PACS numbers: 87.55.km, 87.55.Qr

## I. INTRODUCTION

Patient‐specific quality assurance measurements are commonly performed for IMRT treatments delivered using the TomoTherapy Hi•ART (TomoTherapy, Inc., Madison WI) treatment unit. These measurements are referred to by TomoTherapy as delivery quality assurance (DQA) measurements. Two‐dimensional analysis of the dose distribution is most commonly performed with Kodak EDR2 film (Kodak, Rochester, NY) which saturates above approximately 6 Gy.^(^ ^1^ ^,^ ^2^ ^)^ This implies that to deliver a DQA plan for a treatment fraction which delivers a dose above 6 Gy, the fraction dose would need to be scaled downward in order to make a planar EDR2 film measurement useful. The TomoTherapy treatment planning system (TPS) gives users the option of scaling DQA plans during their creation. This is achieved by decreasing the time that the MLC leaves remain open while other parameters are held constant. This changes the mechanics of the delivery, and raises the question of when a scaled DQA plan adequately mimics the unscaled DQA plan. It is necessary to investigate to what extent the dose for a DQA plan can be scaled before there is a loss of fidelity between the treatment plan and the DQA plan. Measurements for dose scaling factors of 100% to 10% were performed using the PTW 2D‐ARRAY seven29 ion chamber array in the OCTAVIUS phantom (PTW Dosimetry Systems, Freiburg, Germany). The seven29 ion chamber array consists of a 27×27 matrix of ion chambers; each 5 mm×5 mm×5 mm with a center‐to‐center spacing of 1 cm. The useful dose rate is up to 0.5 Gy/sec (0.15 mGy per radiation pulse). Measurements were performed for three patient plans each consisting of four 12 Gy fractions. Film and cylindrical ion chamber measurements were also acquired for one patient plan to compare with the seven29 results.

For fraction sizes larger than 6 Gy, our institution routinely performs two DQA measurements. First, the plan is delivered at the prescription dose and absolute dose measurements are acquired with ion chambers. Second, the DQA plan is scaled to 50% of the prescription dose, and both ion chamber measurements and relative film dosimetry with Kodak EDR2 film are performed. The ratio of the dose measured with the ion chambers to the dose calculated by the TPS is computed and this ratio is here termed the “quality factor”. If the fidelity of the plan remains constant over the necessary range of scaling factors to avoid film saturation (as judged by constancy in the quality factor and ion chamber array measurements), one could then perform only one DQA procedure for treatment plans with doses exceeding 6 Gy.

## II. MATERIALS AND METHODS

Treatment plans for three patients receiving 12 Gy fractions for SBRT of the lung were used to create DQA plans. The DQA plans were scaled between 100% and 10% of the original dose. The PTW seven29 ionization chamber array was used in the PTW OCTAVIUS phantom to obtain measurements. DQA plans were also performed with Kodak EDR2 film in a 30 cm diameter cylindrical Virtual Water phantom (Standard Imaging, Middleton, WI) for Patient 1 for scaling factors 50% to 10% in 10% increments. Ion chamber measurements were performed concurrently with the film measurements using two Exradin A1SL ion chambers (Standard Imaging, Middleton, WI).

Modulation factor (MF) was used as a surrogate for plan complexity, where MF is defined as the longest leaf opening time in a plan divided by the average leaf opening time of all nonzero leaf opening times. The three plans were chosen specifically from our patient database as examples of high, moderate, and low MFs to assess the effects of dose scaling over the range complexities of treatment plans seen clinically. These high, moderate, and low MFs were 1.984, 1.824, and 1.584, respectively. These MFs are consistent with the MF for all tomotherapy plans at our institution. The mean MF for all plans is 1.82 (511 plans), and the mean for 12 Gy SBRT of the lung plans only is 1.88 (12 plans). DQA delivery and analysis were performed according to clinical procedures in use at our institution. VeriSoft (PTW Dosimetry Systems, Freiburg, Germany) Version 4.0 software was used to analyze the seven29 data. The percent gamma pass rates and quality factors for chambers in the high‐dose, low‐gradient region were obtained. Film was digitized on a Vidar scanner (VIDAR Systems Corporation, Herndon, VA) and analyzed with the tomotherapy TPS software. Gamma pass rates, gamma maps, and vertical and horizontal dose profiles were evaluated. The film was additionally analyzed with RIT 113 (Radiological Imaging Technology, Colorado Springs, CO) Version 5.2 software. RIT 113 was used to obtain the percent gamma pass rates comparing the films to the calculated dose distribution from the tomotherapy TPS. Additionally, the 50% scaled film was compared to the films with other scaling factors to more directly show any changes in the radiation delivery due to scaling. That is to say, comparing the films to the standard film (50% scaling factor) would better show changes in the radiation delivery with scaling without the added uncertainty of registering each film to the calculated dose distribution. Alternatively, comparing the films with the calculated dose distribution from the TPS shows the change in the accuracy of the TPS calculations with changes in dose scaling.

The VeriSoft software reports the accumulated dose for the center ion chamber, as well as the ion chamber with the highest dose while a measurement is being performed. Leakage currents (converted to dose) were calculated by observing the total dose accumulated from the time the button to deliver radiation was depressed to the beam on time, typically about one to two minutes.

Mean quality factors of A1SL ion chambers for eleven patients having both 100% and 50% scaled DQA plan results from a clinical database in use at our institution were calculated to supplement the data from the three patients that were examined at scaling factors from 100% to 10%. The eleven patients all received the same 12 Gy per fraction SBRT protocol as the three patient plans that were examined at scaling factors from 100% to 10%. The results from the clinical database were compared with the quality factors obtained with the seven29 for the 100% and 50% scaling factors to show that the three patient plans used to obtain scaling factors from 100% to 10% agree with the results of the larger clinical database for the 100% and 50% scaling factors.

## III. RESULTS

Table 1 reports gamma analysis results for the seven29 data. Table 2 reports the mean and the standard deviation as a percent of the mean (SD (%)) of the quality factors for the three patients individually for the seven29 data. Table 3 reports the mean and the SD (%) for the data from the three patients combined. The 100% scaled DQA plan for Patient 1 was performed after the dose rate for the tomotherapy unit had been adjusted for clinical purposes. This is likely why the mean for the quality factors for the 100% scaled DQA plan for Patient 1 in Table 2 differs from that for the others. The AQF from the clinical database is 1.01±0.01 for the 50% scaled plans and 1.01±0.01 for the 100% scaled plans. Similar to the data from the clinical database, the mean AQF from the seven29 data for the 100% scaled plans agree with the mean AQF for the 50% scaled plan to within one standard deviation, as seen in Tables 2 and 3, once the fact that the measurement for the 100% scaled plan for Patient 1 was obtained after the output had been adjusted is taken into account.

**Table 1:** Percent gamma pass rates for the seven29 data for numbered patient (Pt) plans.

*Scaling Factor (%)*	*Pt 1 (High MF)*	*Pt 2 (Low MF)*	*Pt 3 (Medium MF)*	*Mean for All Patients*	*SD (%) for All Patients* ^*a*^
100	100	99.6	98.9	99.5	0.560
80	100				
60	100				
50	100	99.6	100	99.9	0.231
40	100	100	100	100.0	0.000
30	98.3	100	99.5	99.3	0.880
20	90.3	100	90.4	93.6	5.95
10	46.9	51.4	55	51.1	7.94

a The Standard Deviation (SD) is given as a percentage of the mean.

**Table 2:** Quality factor for the seven29 high‐dose, low‐gradient region for numbered patient (Pt) plans.

*Scaling Factor (%)*	*Center Chamber only for Pt 1*	*Mean for Pt 1 (12 points)*	*SD* ^*a*^ *(%) for Pt 1 (12 points)*	*Center Chamber only for Pt 2*	*Mean for Pt 2 (15 points)*	*SD* ^*a*^ *(%) for Pt 2 (15 points)*	*Center Chamber only for Pt 3*	*Mean for Pt 3 (13 points)*	*SD* ^*a*^ *(%) for Pt 3 (13 points)*
100	0.997	0.999	0.447	1.01	0.998	1.04	0.984	0.993	0.749
50	0.970	0.972	0.234	0.996	0.991	0.768	0.981	0.989	0.867
40	0.972	0.974	0.281	0.994	0.992	0.825	0.983	0.989	0.891
30	0.975	0.975	0.387	0.992	0.991	0.881	0.986	0.990	0.950
20	0.978	0.976	0.620	0.996	0.994	0.792	0.990	0.990	1.16
10	0.953	0.950	1.45	1.01	1.01	2.73	0.961	0.973	2.19

a The Standard Deviation (SD) is given as a percentage of the mean.

**Table 3:** Quality factor mean for all patients combined for the seven29 data.

*Scaling Factor (%)*	*Mean*	*Standard Deviation* ^*a*^ (%)
100	0.997	0.829
50	0.985	1.09
40	0.985	1.08
30	0.986	1.06
20	0.987	1.17
10	0.978	3.22

a The data is for ion chambers in the high‐dose, low‐gradient region. The standard deviation is given as a percentage of the mean.

The A1SL ion chamber results for Patient 1 are reported in Table 4. The tomotherapy TPS analysis of the films shows that the vertical and horizontal profiles exhibit similar trends for any given scaling factor. Specifically, the 10% and 20% scaled DQA plans were lower than the measured doses in the high‐dose, low‐gradient region of the profiles by approximately 3%–10% and 0%–2%, respectively. The 30%–50% scaled DQA plans show measured doses in the high‐dose, low‐gradient region that agree with the calculated values. Since the tomotherapy TPS film analysis doesn't directly give the percentage of passing points for the gamma analysis (only a histogram), RIT 113 was used to obtain this information. Table 5 provides the gamma pass rates using RIT 113 software.

**Table 4:** A1SL ion chamber quality factors.

*Scaling Factor (%)*	*First A1SL Quality Factor*	*Second A1SL Quality Factor*
50	1.01	1.00
40	1.00	0.994
30	1.01	0.991
20	1.02	0.995
10	1.01	1.02

**Table 5:** Film gamma pass rates.

*Film Scaling Factor*	*% Gamma Pass Rate When Compared To Calculated Values from the TPS*	*% Gamma Pass Rate When Compared to the Measured Values from the 50% film*
50%	97%	NA
40%	99%	100%
30%	99%	100%
20%	93%	98.85%
10%	42%	29.71%

**Figure 1:**
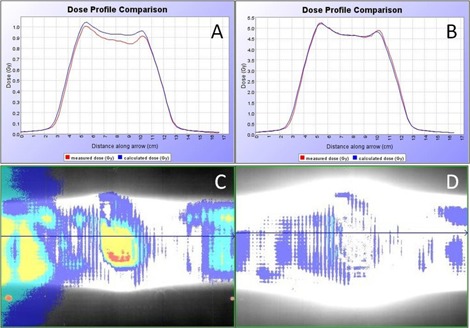
Images from the High•Art TPS software: the panels compare the 10% (A, C) and 50% (B, D) scaled DQA plan vertical profiles and gamma maps for the film analysis for Patient 1. Red and yellow on the gamma maps show points failing gamma analysis (gamma > 1).

**Figure 2:**
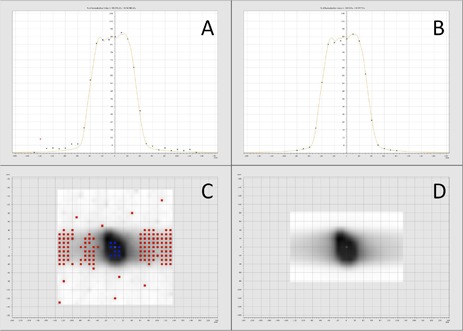
Images from VeriSoft software: the panels compare the 10% (A, C) and 50% (B, D) scaled DQA plan vertical profiles and gamma maps for seven29 analysis for Patient 1. Red and blue on the gamma maps show ion chambers that fail gamma analysis (gamma > 1).

**Figure 3:**
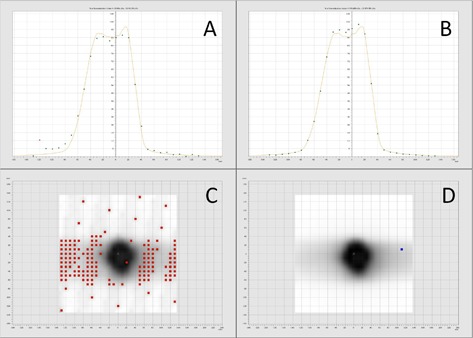
Images from VeriSoft software: the panels compare the 10% (A, C) and 50% (B, D) scaled DQA plan vertical profiles and gamma maps for seven29 analysis for Patient 2. Red and blue on the gamma maps show ion chambers that fail gamma analysis (gamma > 1).

**Figure 4:**
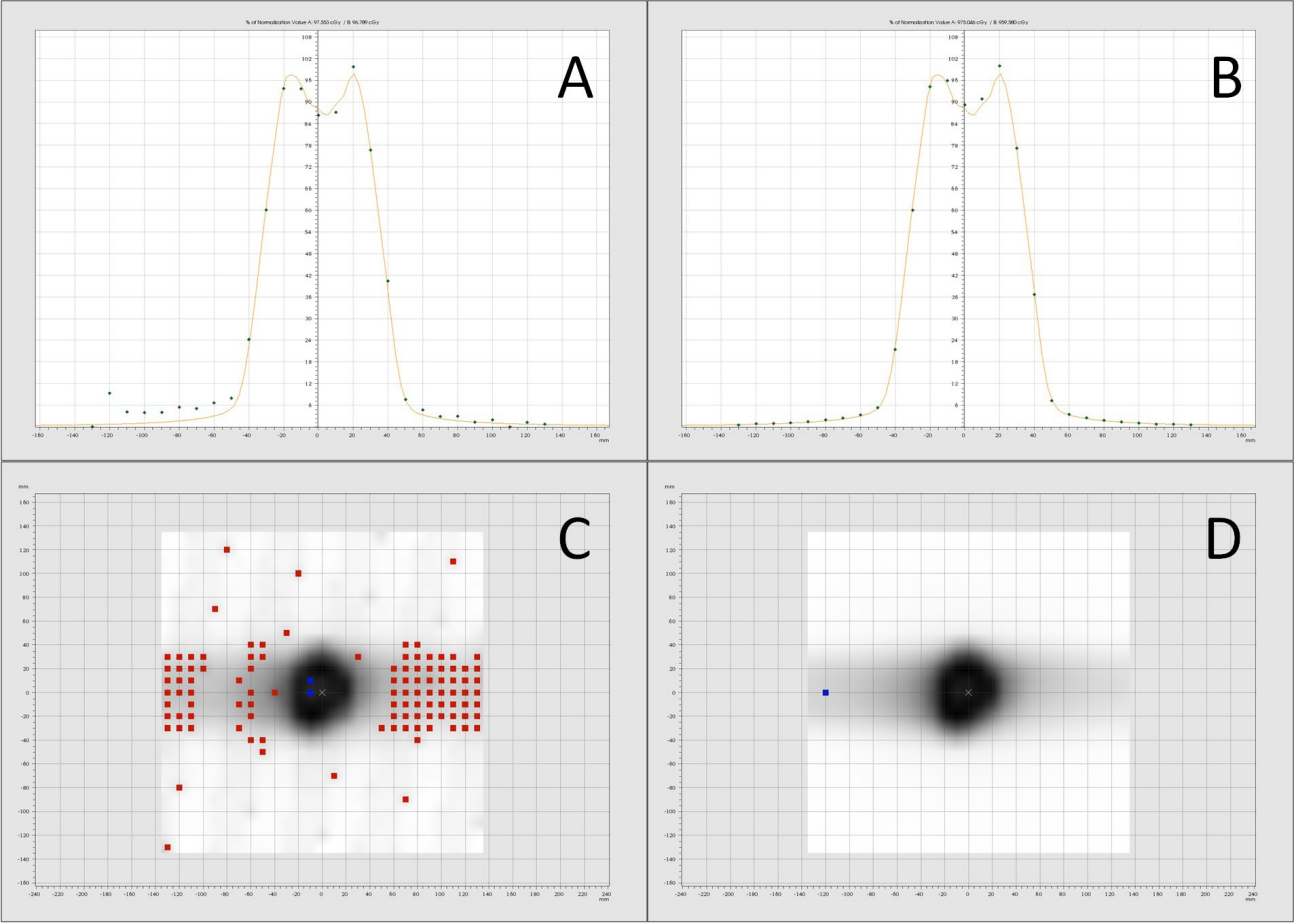
Images from VeriSoft software: the panels compare the 10% (A, C) and 50% (B, D) scaled DQA plan vertical profiles and gamma maps for seven29 analysis for Patient 3. Red and blue on the gamma maps show ion chambers that fail gamma analysis (gamma > 1).

Representative screen images for the tomotherapy TPS film analysis are shown in Fig. 1 for the 10% and 50% scaling factor vertical profiles and gamma maps. Representative screen images for the VeriSoft seven29 analysis are shown in Figs. 2–4 for the 10% and 50% scaling factor vertical profiles and gamma maps. Note that the measured and calculated dose distributions were normalized for all analysis of planar dose distributions for both film and seven29 measurements (i.e., comparison of relative rather than absolute dose distribution data). This was done with the seven29 measurements to assure the analysis was consistent with the film analysis. All AQFs were calculated from absolute dose data.

## IV. DISCUSSION

The gamma pass rates for the seven29 drop from approximately 98%–100% down to approximately 90% for the 20% scaling factor for two of the three patient plans. Even though a gamma pass rate of > 90% is still often considered clinically acceptable, this demonstrates deteriorating agreement with the treatment plan for scaling factors of 20% or less. Twenty percent scaling is also the level at which the film gamma pass rates and profiles first start to show deteriorating agreement with the treatment plan, though again the 20% scaled films are clinically acceptable for plan verification purposes. Since the goal of DQA is to verify the accuracy and fidelity of the delivered treatment plan, not simply to assure that the gamma pass rate threshold isn't exceeded, one shouldn't scale the dose to 20% or less of the prescription treatment dose. The mechanism of scaling the dose could cause a loss of agreement of measured and calculated dose since changing the leaf open times (LOTs), as occurs when dose scaling is done, changes the delivered radiation fluence. The TomoTherapy Hi•ART unit maintains a constant dose rate upstream of the MLCs and intensity modulation is used for all radiation delivery. The length of the LOTs, therefore, determines both the shape of the 3D dose distribution, as well as the amount of radiation delivered (i.e., the dose per fraction to the PTV). When the dose is scaled downward for a DQA plan, LOTs must also be scaled downward. Westerly et al.^(^ ^3^ ^)^ have shown that small LOTs can lead to changes in the quality factor due to the behavior of individual MLC leaves diverging from the behavior modeled by the TPS. The approximation for the mean LOT that Westerly et al. give is reproduced here as Eq. (1), where $t_{\text{mean}}$ is the mean LOT, T is the gantry rotation period, and MF is the modulation factor:

(1) $$t_{\text{mean}}\approx \left(1/\text{MF}\right)\left(T/51\right)$$

Westerly et al. state that the poor agreement between measured and calculated dose they observed occurs mainly with mean LOTs below 100 msec. Using the above equation to estimate the mean LOTs for the unscaled DQA plans, Patients 1, 2, and 3 have mean LOTs of 514, 681, and 473 msec, respectively. Assuming the mean LOT for scaled DQA plans is just the unscaled mean LOT times the scaling factor, the mean LOTs are 103, 136, and 95 msec at 20% dose scaling for Patients 1, 2, and 3, respectively, and are all below 100 msec at 10% dose scaling. That the DQA plan quality deteriorates for scaling factors whose mean LOTs are at or below 100 msec seems suggestive. One possible concern with the assumption that the scaled mean LOT is the unscaled LOT times the scaling factor is if the tomotherapy unit doesn't deliver a projection for a leaf pair due to the LOT dropping below some threshold value. That is to say, instead of that LOT being the unscaled LOT times the scaling factor, it is just forced to zero as Thomas et al.^(^ ^4^ ^)^ state can happen when scaling is performed. This could alter the delivered fluence, especially for highly scaled DQA plans.

It is not clear that a different treatment protocol would show results that deteriorate at the same scaling factor as a 12 Gy SBRT fraction, so it is advisable that any new DQA protocol that calls for dose scaling to be carefully studied to assure agreement with the original plan. It seems likely that part of the loss of agreement between measured and calculated dose is due to the deteriorating signal to noise ratio (SNR). Additionally, since dose scaling is achieved by changing the LOTs but not the time over which radiation is delivered, the relative effects of tongue‐and‐groove leakage radiation would be more severe if the tomotherapy TPS isn't able to completely account for this leakage radiation. This would further lower the SNR for smaller scaling factors since the signal would be smaller, while that part of tongue‐and‐groove leakage radiation that the TPS didn't account for remained constant. One might consider the SNR for comparing different scaled DQA protocols. The SNR can change for different size fractions. Consider the simplified case of the signal being the prescribed dose per fraction (i.e., the dose to the PTV) and the noise as the leakage dose (the part of the measured dose due only to leakage current) times the total treatment time. The SNR of a scaled plan is the SNR of the unscaled plan times the scaling factor, since the treatment time remains the same and the dose delivered is reduced to the scaling factor times the prescription dose. The 12 Gy fractions for this research were about 17 minutes long, and the average leakage current (converted to dose) was about $4\;\times \;10^{-5}\;\text{Gy}/\text{sec}$. Using these parameters, the SNR is found to be 294 for a 12 Gy DQA plan. A 2 Gy fraction with the same leakage dose rate and the average treatment time of 4.8 minutes seen at our institution gives a SNR of 174. The SNRs of 294 and 174 for the unscaled plans imply that the measured charge due to ionization alone would be increased by 0.34% and 0.57%, respectively, due to the leakage current. Both values are acceptably small, however, because as one scales to 10% of the treatment dose, the leakage current will then increase the measured charge by 3.4% and 5.7%, respectively. Using the above assumptions, scaling the 12 Gy DQA plan to 100%, 50%, 40%, 30%, 20%, and 10% implies that the noise increases the measured dose due to ionization alone by 0.34%, 0.68%, 0.85%, 1.1%, 1.7%, and 3.4%, respectively. Considering all of the other uncertainties involved, allowing the collected charge to be increased by 1.7% or 3.4% due to noise is unacceptable. Thus, there could be clinically relevant differences in SNRs for scaled plans even though the SNRs for the unscaled plans show no clinically relevant differences. The example SNR for the 2 Gy DQA plan is illustrative only, since dose scaling isn't necessary at 2 Gy. It does, however, show that different treatment protocols can exhibit different SNRs; therefore, it is likely that other treatment protocols that require dose scaling for DQA plans would show SNRs different from the treatment plans considered in this paper. In light of this fact, one should perform comparisons similar to those outlined in this paper to ensure that the scaling of the dose does not significantly alter the ability of the scaled DQA plan to model the treatment plan. In the absence of an ion chamber array, radiochromic film might be considered as an alternative for obtaining planar dose distribution data for unscaled DQA plans that would otherwise saturate silver‐halide film.

A final point of discussion is that the dose for a scaled DQA plan is scaled downward by decreasing the LOTs while the pitch, dose rate (upstream of the MLC's), projection interval, and other relevant treatment parameters are held constant. This implies that the delivered fluence changes due to the change in the angular distribution of the fluence. For example, there are 51 projection intervals per rotation, so each projection occurs over about seven degrees of gantry rotation. If the unscaled DQA plan had an LOT equal to the total projection time for a given projection interval, the LOT in the 10% scaled DQA plan would now only occupy 10% of the projection interval, or about 0.7 degrees of rotation. This changes the dose distribution by changing the angle at which the radiation arrives at a point in the phantom. The amount of material that is exposed to the beam for a projection interval is also changed due to the radiation arriving through a smaller subarc of the gantry rotation. The geometry of the system could change the effective depth that the radiation from a given LOT has to pass through in order to reach a point at depth. A cylinder that was coaxial to the gantry rotation would have the same average depth to the isocenter regardless of the scaling factor, since every gantry angle has the same source‐to‐surface distance (SSD). The OCTAVIUS phantom in a coaxial geometry with an LOT starting when the source and one vertex of the octagonal phantom and the isocenter line up would give relative depths to the isocenter of 0.978, 0.988, and 0.997 (taking a depth of 1.00 to equal the depth from a vertex of the phantom to the isocenter) for the unscaled, 50% scaled, and 10% scaled DQA plans, respectively. While this is an idealized case, it does illustrate that care must be taken when using any noncylindrical, noncoaxial phantom for running scaled DQA plans. The OCTAVIUS phantom's geometry could be partly responsible for the poor agreement between unscaled and highly‐scaled DQA plans seen in the seven29 data.

## V. CONCLUSIONS

One should use caution when scaling any DQA plan. The results presented here show that scaling too much can lead to loss of agreement between the scaled and unscaled DQA plan, as well as with the original treatment plan the QA is intended to verify. Any time a new QA protocol is created that involves dose scaling for DQA plans, it is advisable to test some representative plans to ensure agreement with unscaled DQA plans. SNRs may be useful in determining if a DQA plan is scaled too much. One should keep in mind that the delivered fluence will change compared to the patient treatment, since the dose is decreased by decreasing the LOTs while holding other parameters constant. The effect increases as dose is scaled to smaller fractions of the prescribed treatment dose. Thus, one should scale the dose to the smallest degree possible in order to mimic the patient treatment delivery as closely as possible. Analysis of the dose distribution using many measurement points in two dimensions is necessary since DQA plans with small scaling factors show significant discrepancies in the measurements using the seven29 array and film, yet these discrepancies were not observed for two‐point measurements alone in the PTV.

The results show that it is sufficient to perform the 50% scaled DQA plan created with our institution's clinical protocols alone with both film and A1SL ion chambers, negating the need to perform a second DQA scaled at 100%.
